# Changes in the Pulmonary Function Test after Radioactive Iodine Treatment in Patients with Pulmonary Metastases of Differentiated Thyroid Cancer

**DOI:** 10.1371/journal.pone.0125114

**Published:** 2015-04-29

**Authors:** Eun Kyung Jang, Won Gu Kim, Ho-Cheol Kim, Jin-Won Huh, Hyemi Kwon, Yun Mi Choi, Min Ji Jeon, Tae Yong Kim, Young Kee Shong, Jin-Sook Ryu, Won Bae Kim

**Affiliations:** 1 Division of Endocrinology and Metabolism, Department of Internal Medicine, Asan Medical Center, University of Ulsan College of Medicine, Seoul 138–736, Korea; 2 Division of Pulmonology and Critical Care Medicine, Department of Internal Medicine, Asan Medical Center, University of Ulsan College of Medicine, Seoul 138–736, Korea; 3 Department of Nuclear Medicine, Asan Medical Center, University of Ulsan College of Medicine, Seoul 138–736, Korea; 4 Division of Endocrinology, Dongnam Institute of Radiological and Medical Sciences Cancer Center, Busan, Korea; Tel Aviv Sourasky Medical Center, ISRAEL

## Abstract

**Objective:**

Pulmonary function test (PFT) is a useful tool for an objective assessment of respiratory function. Impaired pulmonary function is critical for the survival and quality of life in patients with pulmonary metastases of solid cancers including thyroid cancer. This study aimed to evaluate clinical factors associated with severely impaired pulmonary function by serial assessment with PFT in patients with pulmonary metastasis of differentiated thyroid cancer (DTC) who received radioactive iodine treatment (RAIT).

**Patients:**

This retrospective study enrolled 31 patients who underwent serial PFTs before and after RAIT for pulmonary metastasis of DTC. We evaluated the risk factors for severe impairment of pulmonary function.

**Results:**

The median age of the patients was 44.1 years and 18 of them were female patients. Severe impairment of pulmonary function was observed in five patients (16%) after a median of three RAITs (cumulative I-131 activity = 20.4 GBq). These patients were older and more frequently had mild impairment of baseline pulmonary function, respiratory symptoms, or progressive disease compared with patients with stable pulmonary function. Neither cumulative dose nor number of RAIT was associated with decreased pulmonary function. Coexisting pulmonary diseases, presence of respiratory symptoms, and metastatic disease progression were significantly associated with severe decrease in forced vital capacity during follow-up (*p* =.047, *p* =.011, and *p* =.021, respectively).

**Conclusions:**

Pulmonary function was severely impaired during follow-up in some patients with pulmonary metastasis of DTC after a high-dose RAITs. Neither the number of RAIT nor the cumulative I-131 activity was associated with decreased pulmonary function. Serial PFT might be considered for some high-risk patients during follow-up.

## Introduction

Pulmonary function test (PFT) is a useful tool for objective assessment of respiratory impairment [1, 2]. Serial PFTs may be crucial for tracking pulmonary diseases, quantifying responses to therapy, and early diagnoses of lung injury after exposure to chemical dusts or radiation therapy [1]. Cardiopulmonary function is an important predictor of survival in patients with metastatic breast cancer [3]. Impairment of pulmonary function could be more important for quality of life in patients with pulmonary metastases of differentiated thyroid cancer (DTC) due to its relatively longer survival period compared with other cancers. Therefore, it is important to know the risk factors for worsening pulmonary function in patients with pulmonary metastases of DTC.

Radioactive iodine treatment (RAIT) is an effective therapeutic modality for DTC patients with distant metastases [4, 5]. The lung is the most common metastatic site of DTC. Radioactive iodine refractoriness is an important prognostic factor in patients with metastatic DTC. About 70% of pulmonary metastases of DTC are radioactive iodine-avid, and their 10-year survival rates are about 60%. However, those with radioactive iodine refractory metastases of DTC have only 10% of 10-year survival rate [6–9].

Radiation-induced pulmonary fibrosis was reported in 1–7% of patients with pulmonary metastases of DTC after RAIT [10–12]. The risk of pulmonary toxicity is increased when multiple RAITs are administered in a short interval or high activities of radioactive iodine are administered to patients with diffuse iodine-avid pulmonary metastases [10]. Most RAIT-induced pulmonary toxicity has been reported in children [11, 13], and only a few studies have reported on RAIT-induced pulmonary toxicity in adult patients with pulmonary metastases of DTC [12, 14]. No report has evaluated serial PFTs before and after RAIT to elucidate the risk factors for impairment of pulmonary function in these patients [14].

In this study, we evaluated the changes in serial PFTs in patients with pulmonary metastases of DTC during a median 4.1-year follow-up. We also evaluated the clinical features associated with impaired pulmonary function after RAIT in these patients.

## Materials & Methods

### Patients

We retrospectively reviewed patients who were diagnosed with pulmonary metastases from DTC and underwent RAIT between 1995 and 2012 in Asan Medical Center, Seoul, Korea. Thirty one adult patients were enrolled in whom PFT was carried out before RAIT and more than once after RAIT. This study protocol was approved by the institutional review board of Asan Medical Center (IRB number: 2014–0511). Informed consent was waived due to the retrospective nature of this study and patient records or information was anonymized prior to analysis.

### Diagnosis of pulmonary metastases

The diagnosis of pulmonary metastases was confirmed by clinical assessment, including I-131 uptake in the lung using an I-131 whole-body scan (WBS) following RAIT, chest X-ray, chest computed tomography (CT), 18F-fluorodeoxyglucose-positron emission tomography with CT (PET-CT), and/or pathological examination of the pulmonary metastatic site. Images of WBS were reviewed by two experienced nuclear medicine specialists. Abnormal I-131 uptake in WBS was further evaluated with other radiologic images such as chest X-ray, chest CT, or PET-CT. Nineteen patients had confirmed pulmonary metastases before RAIT in other radiologic images including chest X-ray, chest CT, or PET-CT. Twenty-six patients exhibited abnormal pulmonary uptake in post-ablative WBS and 12 patients were clinically diagnosed with pulmonary metastases only according to the imaging findings of the WBS. Two patients underwent a lung biopsy and pulmonary metastases were confirmed by histopathology.

### Treatment and follow-up protocols

A high fixed dose of I-131 (7.4 GBq, 200 mCi) was administered to patients who were diagnosed with pulmonary metastases before the initial RAIT. All the patients who were diagnosed pulmonary metastases after RAIT received 5.55 GBq (150 mCi) as an initial therapy. All patients underwent high-dose I-131 treatment after thyroxine withdrawal for 5 to 6 weeks, and post-therapeutic WBS (RxWBS) images were obtained at 2 days and 5 to 7 days after administration of I-131. All patients were treated with thyroxine to suppress thyroid-stimulating hormone and were regularly followed up with physical examination and serum thyroglobulin (Tg) and/or diagnostic imaging studies every 6 months [15].

### Definition of clinical parameters

We defined coexisting pulmonary disease as a history of pulmonary disease and structural pulmonary lesions on radiologic imaging consistent with this history. Respiratory symptoms were defined as newly developed dyspnea after RAIT. We categorized the radioactive iodine uptake pattern on WBS according to the timing of the appearance of diffuse pulmonary uptake. Diffuse uptake at 2 days after RAIT was defined as early diffuse uptake on WBS and diffuse uptake at 7 days after RAIT was defined as delayed diffuse uptake on WBS. For patients with macronodular pulmonary lesions (larger than 1 cm along the longest diameter of the tumor), disease status was classified using the Response Evaluation Criteria in Solid Tumors (RECIST; version 1.1).

Because RECIST cannot be applied to patients with micronodular pulmonary lesions (less than 1 cm along the longest diameter of the tumor), we used the disease status criteria from a previous study of these patients [16]. Briefly, patients with no lesions visible on radiologic imaging and undetectable stimulated Tg levels (less than 1.0 μg/L) were defined as complete response (CR). The response was defined as a partial response (PR) if stimulated or suppressed Tg levels were detectable but without progressive elevation, the size of a pulmonary metastatic lesion of 3 mm or more in size had decreased, and/or one or more lung lesion had disappeared. Stable disease (SD) was defined as a metastatic lesion with less than a 3-mm change in size, no change in the number of pulmonary nodules, and detectable stimulated or suppressed Tg levels without progressive elevation of Tg. Progressive disease (PD) was defined as increasing stimulated or suppressed Tg levels, new metastatic foci detected on radiologic imaging, and/or a 3-mm or more increase in the size of any pulmonary lesions.

### Pulmonary function tests

Spirometry was performed as recommended by the American Thoracic Society (Vmax 22, SensorMedics; PFDX, MedGraphics, St Paul, Minn) [17]. Forced vital capacity (FVC), forced expiratory volume in 1 second (FEV1), and FEV1/FVC were expressed as the percentage of measured to predicted values. Carbon monoxide diffusing capacity (DL_CO_) was measured by the single-breath method using a Vmax 229D (SensorMedics) or a Masterlab Body (Jaeger AB) [18]. The predicted values of FEV1, FVC, FEV1/FVC, and DL_CO_ were calculated from Korean equations formulated using data of a non-smoking population. This equation adjusts predicted value for age, sex, height, and weight [19].

PFTs were usually performed when patients complained of respiratory symptoms, or sometimes as preoperative work-up. All patients in this study underwent PFT at least once before RAIT. Abnormal pulmonary function was classified as a restrictive pattern or obstructive pattern. A restrictive pattern was defined as an FVC of less than 80% with an FEV1/FVC of 70% or more. Restrictive patterns were subcategorized as mild (FVC of 60% or more), moderate (FVC of less than 60%), and severe (FVC of 50% or less). An obstructive pattern was defined as an FEV1/FVC of less than 70% and an FVC of 80% or more. Obstructive patterns were subcategorized as mild (FEV1 of 60% or more), moderate (FEV1 of less than 60%), and severe (FEV1 of 40% or less) [20]. We defined a significant decrease in pulmonary function as a severe restrictive or obstructive pattern.

### Statistical analysis

R version 3.0 and the R libraries prodlim, car, Cairo, and survival were used for analyzing data and drawing graphs (R Foundation for Statistical Computing, Vienna, Austria; http://www.R-project.org). Continuous variables between two groups were compared using a Student’s *t*- test or Wilcoxon rank-sum test. Categorical variables between two groups were compared using a chi-squared test or Fisher exact test. Variables among patients with normal pulmonary function, restrictive patterns, and obstructive patterns were analyzed using a Kruskal-Wallis test. Pulmonary function changes before and after RAIT within subjects were assessed by a paired *t*-test or Wilcoxon signed-rank test. Simple linear regression analysis was used to compare the changes in pulmonary function during follow-up according to the clinical parameters. We used the multiple linear regression analysis to find variables associated with change of FVC. Multiple linear regression analysis included factors associated with decreased FVC in single linear regression analysis. Significant factors of simple linear regression analysis (respiratory symptom, coexisting pulmonary disease, metastases pattern on chest X-ray, and progressive disease) were included in multiple linear regression analysis. Respiratory symptom and metastases pattern on chest X-ray were excluded in the process of analysis. All *p* values were two sided, with a *p* value <. 05 considered statistically significant.

## Results

### Baseline characteristics and PFT values of patients

The median patient age (n = 31) was 44.1 years old and 18 patients (58%) were female (Table 1). Thirty patients (97%) were diagnosed with papillary thyroid carcinoma (PTC) and one patient (3%) had follicular thyroid carcinoma (FTC). Five patients (16%) had coexisting pulmonary disease, including pulmonary tuberculosis, interstitial lung disease, bronchiectasis, and emphysema. Pulmonary metastases were confirmed in 19 patients (61%) before RAIT and pulmonary metastases were diagnosed in 12 patients (39%) after initial RAIT. The median cumulative I-131activity was 20.35 GBq (550mCi) and patients received RAITs a median of three times during follow-up. Macronodular pulmonary metastases were found in 11 patients (35%). Diffuse radioactive iodine uptake patterns on WBS were found in 24 patients (77%) and 12 patients (39%) demonstrated disseminated pulmonary metastases on chest X-ray or chest CT. Eight patients (26%) had progressive pulmonary metastases (progressive disease) and five patients (16%) died during follow-up.

**Table 1:** Baseline Characteristics of Patients.

	Normal pulmonary function (n = 21)	Restrictive (n = 7)	Obstructive (n = 3)	*p* ^*a*^	*p* ^*b*^
Sex, female	11 (52)	5 (71)	2 (67)	.45	.65
Age at first RAIT (year), median (IQR)	39.4 (28.2, 60.9)	46.4 (26.0, 59.5)	47.0 (46.6, 59.5)	.6	.53
Age at first RAIT ≥ 45 years	8 (38)	4 (57)	3 (100)	.14	.12
Baseline pulmonary function (%^†^), median (IQR)					
FVC	91.0 (87.0, 95.0)	71.0 (68.0, 76.5)	97.0 (95.0, 104.5)	.042	<. 001
FEV1	96.0 (91.0, 103.0)	79.0 (71.0, 84.0)	78.0 (74.5, 78.0)	<. 001	<. 001
FEV1/FVC	84.0 (80.0, 89.0)	86.0 (82.5, 90.5)	64.0 (54.0, 66.5)	.5	.015
DL_CO_ ^*^	103.0 (94.8, 108.5)	81.0 (76.3, 86.5)	63.0 (NA)	.012	.025
Tumor size (cm), median (IQR)	3.0 (2.0, 4.5)	5.0 (3.0, 6.3)	5.0 (3.9, 5.0)	.13	.29
T & N staging^**^					
T1/2	2 (10)	1 (14)	0 (0)	.99	.79
T3/4	19 (91)	6 (85)	3 (100)	-	-
pN1a/N1b	20 (95)	7 (100)	3 (100)	.99	.79
Respiratory symptoms	6 (29)	3 (43)	2 (67)	.42	.4
Coexisting pulmonary disease	3 (14)	1 (14)	1 (33)	.99	.7
Size of metastatic lesion					
Micronodular metastases	14 (67)	4 (57)	2 (67)	.99	.9
Macronodular metastases	7 (33)	3 (43)	1 (33)	-	-
Disseminated pattern on chest CT	8 (38)	3 (43)	1 (33)	.99	.96
Diffuse uptake on WBS	16 (76)	6 (86)	2 (68)	.99	.79
Early diffuse uptake	8 (38)	5 (71)	1 (34)	.44	.29
Delayed diffuse uptake	8 (38)	1 (15)	1 (34)	-	-

^a^ *p-*value comparing normal pulmonary function and abnormal pulmonary function groups.

^b^ *p-*value according to one-way ANOVA comparing patients with normal pulmonary function, restrictive patterns, and obstructive patterns.

^†^ % in FVC, FEV1, and DL_CO_ denote percentage of measured to predicted values.

^*^DL_CO_ was measured in 12 patients with normal pulmonary function, 4 patients with a restrictive pattern, and 1 patient with an obstructive pattern.

^**^Tumor and lymph node staging according to the TNM staging system (American Joint Committee on Cancer, Seventh Edition, 2010).

n, number; RAIT, radioactive iodine treatment; IQR, inter-quartile range; FVC, forced vital capacity; FEV1, forced expiratory volume in 1 second; DL_CO_, Carbon monoxide diffusing capacity; NA, not applicable; CT, computed tomography; WBS, whole body scan.

At baseline PFTs, 21 patients (68%) had normal pulmonary function, whereas 10 patients had abnormal pulmonary function including 7 patients (23%) with a mild restrictive pattern and 3 patients (10%) with a mild obstructive pattern. No patient had a moderate to severe restrictive or obstructive pattern at baseline PFTs. There were no differences in age, sex, tumor size, T staging, N staging, respiratory symptoms, coexisting pulmonary disease, detection time of metastases, radioactive iodine uptake pattern on WBS, and the metastatic pattern on chest X-ray or chest CT between the normal and abnormal baseline PFT groups (Table 1).

### Serial changes in PFTs after RAIT according to the baseline pulmonary function

**Fig 1:**
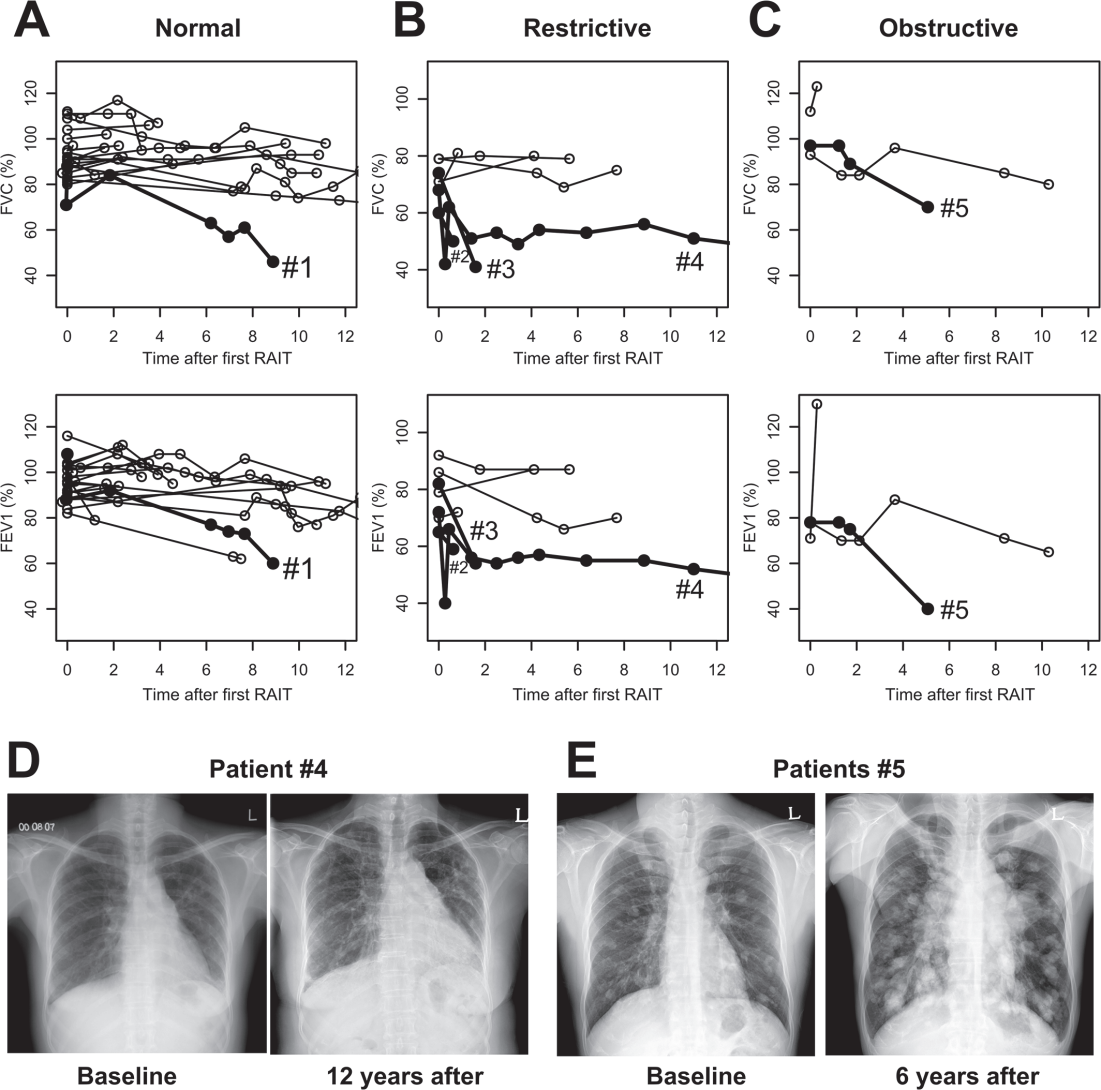
Serial changes in pulmonary function during follow-up after RAIT in patients with pulmonary metastases of differentiated thyroid cancer. Patients were classified according to their baseline pulmonary function. We entered the values of baseline PFT at the time of the first RAIT (time ‘0’) into this figure. (A) Changes in FVC and FEV1 in patients with normal pulmonary function at baseline (n = 21). (B) Changes in FVC and FEV1 in patients with restrictive pulmonary function at baseline (n = 7). (C) Changes in FVC and FEV1 in patients with obstructive pulmonary function at baseline (n = 3). The bold lines indicate the patients who demonstrated a significantly decreased pulmonary function (severe restrictive or obstructive pattern) during follow-up. Each number of the bold lines corresponds to the patient number as shown in Table 2. (D) Patient #4 had mild interstitial lung disease, and chest radiography before RAIT demonstrated ill-defined patch reticular opacities in sub-pleural areas. About 12 years after the first RAIT, her chest radiography showed an increased extent of multifocal patch reticular and ground-glass opacities in peripheral areas of both lungs compared with chest radiography before RAIT. (E) Patient #5 was diagnosed with pulmonary metastases before thyroid surgery and 1-2cm sized multiple metastatic nodules were seen in both lungs on chest radiography before RAIT. The size of the metastatic nodules was increased in follow-up chest radiography even after high-dose RAITs. %, % of measured to predicted values. RAIT, radioactive iodine treatment; PFT, pulmonary function test; FVC, forced vital capacity; FEV1, forced expiratory volume 1 second.

The median time interval between the first RAIT and last PFT was 4.1 years [inter-quartile range (IQR), 1.7–9.9]. The median values of the worst pulmonary function during follow-up were as follows: FVC = 87.0%, FEV1 = 85.0%, FEV1/FVC = 79.0% and DL_CO_ = 82.0%. In 26 of 31 patients (84%), there were no significant changes in serial PFTs during follow-up. However, five patients (16%) had a significant decrease in their pulmonary function (severe restrictive or obstructive pattern) during follow-up as shown in Fig 1 and Table 2.

**Table 2:** Patients with Severe Impairment of Pulmonary Function During Follow-up after RAIT.

Patient number	Patient #1	Patient #2	Patient #3	Patient #4	Patient #5
Sex	M	F	M	F	M
Age at first RAIT (years)	71	64	62	46	46
Coexisting pulmonary disease	present	none	none	present	none
Baseline pulmonary function					
FVC (%^*^)	88	60	74	68	97
FEV1 (%^*^)	108	65	82	72	78
FEV1/FVC (%)	84	77	83	82	69
DL_CO_ (%^*^)	108	None	83	79	63
Worst pulmonary function during f/u					
FVC (%^*^)	46	50	41	42	70
FEV1 (%^*^)	60	59	54	40	40
FEV1/FVC (%)	73	84	100	74	47
DL_CO_ (%^*^)	45	78	45	55	55
Detection time of metastases	After RAIT	Before RAIT	Before RAIT	After RAIT	Before RAIT
Cumulative I-131 activity (GBq)	20.35	7.4	14.8	20.35	14.8
Serum Tg at first RAIT (μg/L)	25	2	9720	60.4	4620
Uptake pattern on WBS	Focal	Diffuse	Diffuse	Diffuse	Diffuse
Metastases pattern on chest CT	Focal	Disseminated	Disseminated	Focal	Disseminated
Disease status	PD	PD	PD	CR	PD
Survival	Alive	Expired	Alive	Alive	Alive

%^*^, % in FVC, FEV1, and DL_CO_ denotes percentage of measured to predicted values.

RAIT, radioactive iodine treatment; M, male; F, female; FVC, forced vital capacity; FEV1, forced expiratory volume in 1 second; DL_CO_, Carbon monoxide diffusing capacity; f/u, follow-up; Tg, thyroglobulin; WBS, whole body scan; CT computed tomography; PD, progressive disease; CR, complete response.

### Patients with normal pulmonary function at baseline

Baseline PFT was normal in 21 of 31 patients (68%). One patient had a significant decrease in pulmonary function during follow-up (Patient #1; Fig 1A). The patient was treated for pulmonary tuberculosis 12 years before the thyroid surgery. In the chest radiography before RAIT, there was a small volume loss with fibrotic changes in both upper lobes but his baseline PFT was normal. The patient had pulmonary metastases with focal radioactive iodine uptake on RxWBS and underwent two more high-dose RAITs. Metastatic pulmonary lesion of the patient progressed and pulmonary function was aggravated during follow-up as shown in Fig 1A.

### Patients with restrictive pulmonary function at baseline

Baseline PFTs of seven patients (23%) showed a mild restrictive pattern. Three patients (43%) had significant impairment in their pulmonary function during follow-up (Patients #2, #3, and #4; Fig 1B). Patients #2 and #3 had no pulmonary disease before RAIT. Their metastatic diseases were disseminated in both lungs on chest CT and there was diffuse radioactive iodine uptake on WBS. The metastatic diseases of these two patients were radioactive iodine refractory and progressive after RAITs. Patient #2 expired during follow-up due to disease progression.

Patient #4 had a mild interstitial lung disease and the baseline PFT showed a mild restrictive pattern. There were ill-defined patchy reticular opacities in sub-pleural areas on chest radiography before RAIT (Fig 1D). In the RxWBS, there was diffuse RAI uptake in both lung fields, and the patient underwent two more high-dose RAITs. All metastatic lesions had disappeared on imaging studies and her serum Tg was undetectable after three RAITs. The patient had lived disease-free for 13 years of follow-up since the last RAIT. However, the pulmonary function was impaired to a severe restrictive pattern from1 year after the first RAIT. There were increased fibrotic changes in both lung fields on the chest radiography suggesting aggravated interstitial lung disease (Fig 1D).

### Patients with obstructive pulmonary function at baseline

Baseline PFTs of three patients showed a mild obstructive pattern. One patient had a significant decrease in his pulmonary function during follow-up (Patient #5; Fig 1C). Pulmonary metastases of FTC were confirmed before thyroid surgery in this patient. There were multifocal macronodular pulmonary metastases on preoperative chest radiography (Fig 1E). He did not have any coexisting pulmonary disease. Metastatic pulmonary disease was progressive even after two high-dose RAITs and he was managed with watchful waiting. His pulmonary function deteriorated to a severe obstructive pattern during follow-up due to progressive metastatic disease that compressed the airway.

### Clinical factors associated with impairment of pulmonary function after RAIT

We categorized five patients (Patients #1-#5) who had a severe restrictive or obstructive pattern in serial PFTs as an "impaired PFT group" and the remaining 26 patients as a "stable PFT group" (Table 3). The age at first RAIT was higher in the impaired PFT group than in the stable PFT group (median 62.1 vs. 38.3 years, *p =*. 028). Mild impairment of baseline pulmonary function was more frequent in the impaired PFT group [odds ratio (OR) = 12.02, *p =*. 027]. The impaired PFT group had more respiratory symptoms than the stable PFT group (OR = 9.94, *p =*. 042), and progressive diseases were also more frequent in the impaired PFT group (OR = 18.91, *p =*. 01).

**Table 3:** Clinical Features of Patients According to Pulmonary Function During Follow-up.

	Stable PFT group (n = 26)	Impaired PFT group (n = 5)	*p*
Sex, female	16 (62)	2 (40)	.63
Age at first RAIT (year), median (IQR)	38.3 (28.1. 59.2)	62.1 (46.4, 64.7)	.028
Age at first RAIT ≥ 45 years	10 (38)	5 (100)	.018
Patients with mild impairment of baseline pulmonary function	6 (23)	4 (80)	.027
Baseline pulmonary function (%^†^), median (IQR)			
FVC	90.5 (82.3, 94.8)	74.0 (68.0, 88.0)	.12
FEV1	93.5 (86.5, 100.2)	78.0 (72.0, 82.0)	.2
FEV1/FVC	84.0 (80.0, 89.0)	82.0 (77.0, 83.0)	.18
DL_CO_ *	101.0 (91.0, 106.0)	81.0 (75.0, 89.3)	.21
Worst pulmonary function (%^†^), median (IQR)			
FVC	90.0 (80.3, 96.8)	46.0 (42.0, 50.0)	<. 001
FEV1	90.0 (77.5, 95.0)	54.0 (40.0, 59.0)	<. 001
FEV1/FVC	79.5 (75.0, 82.8)	74.0 (73.0, 84.0)	.72
DL_CO_ *	88.0 (80.0, 96.0)	55.0 (52.5, 60.8)	.01
Tumor size (cm), median (IQR)	3.2 (2.1, 4.9)	5.0 (3.0, 6.5)	.3
T & N staging**			
T1/2	3 (11)	0 (0)	.99
T3/4	23 (88)	5 (100)	-
pN1a/N1b	25 (96)	5 (100)	.99
Respiratory symptoms	7 (27)	4 (80)	.042
Coexisting pulmonary disease	3 (12)	2 (40)	.17
Number of RAITs, median (IQR)	3 (2, 4)	2 (2, 3)	.98
Cumulative I-131 activity (GBq), median (IQR)	22.2 (13.41, 27.75)	14.8 (14.8, 20.35)	.18
Size of metastatic lesion			
Micronodular metastases	18 (69)	2 (40)	.32
Macronodular metastases	8 (31)	3 (60)	-
Disseminated pattern on chest CT	9 (35)	3 (60)	.35
Diffuse lung uptake on WBS	20 (76)	4 (80)	.99
Early diffuse uptake	10 (38)	4 (80)	.15
Delayed diffuse uptake	10 (38)	0 (0)	-
Progressive disease, n (%)	4 (15)	4 (80)	.01
Death, n (%)	4 (15)	1 (20)	.99

All patients with decreased lung function demonstrated either a severe restrictive pattern (four patients) or a severe obstructive pattern (one patient).

^†^%, % in FVC, FEV1, and DL_CO_ denote percentage of measured to predicted values.

^*^Baseline DL_CO_ was measured in 13 patients with stable pulmonary function and 4 patients with decreased pulmonary function. DL_CO_ was measured during follow-up in 20 patients in the stable PFT group and 4 patients in the impaired PFT group.

^**^Tumor and lymph node staging according to the TNM staging system (American Joint Committee on Cancer, Seventh Edition, 2010).

RAIT, radioactive iodine treatment; IQR, inter-quartile range; FVC, forced vital capacity; FEV1, forced expiratory volume in 1 second; DL_CO_, Carbon monoxide diffusing capacity; CT, computed tomography; WBS, whole body scan.

### Clinical factors to predict impairment of pulmonary function

#### Coexisting pulmonary disease

**Fig 2:**
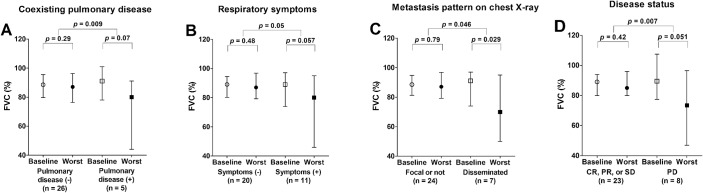
Clinical factors associated with severe impairment of pulmonary function after radioactive iodine treatment. (A) Changes in FVC at baseline and the worst value during follow-up in patients with or without coexisting pulmonary disease. (B) Changes in FVC at baseline and the worst value during follow-up in patients with or without respiratory symptoms. (C) Changes in FVC at baseline and the worst value during follow-up in patients with or without disseminated metastases on chest X-ray (D) Changes in FVC at baseline and the worst value during follow-up in patients with or without progressive disease. %, % of measured to predicted values. FVC, forced vital capacity; Baseline, pulmonary function at baseline; Worst, worst values of pulmonary function during follow-up; CR, complete response; PR, partial response; SD, stable disease; PD, progressive disease.

The presence of coexisting pulmonary disease was significantly associated with decreased FVC and FEV1 in serial PFTs (Fig 2A). The median FVC at baseline PFT in patients with coexisting pulmonary disease (n = 5) was 91.0%, which decreased to 80.0% during follow-up after RAIT (*p =*. 07). In patients without coexisting pulmonary disease (n = 26), the median FVC changed from 88.5% to 87.0% during follow-up (*p =*. 29).The decrease in the FVC was more significant in patients with coexisting pulmonary disease than in those without coexisting pulmonary disease (*p =*. 009; Fig 2A and S1 Table). FEV1significantly decreased during follow-up in patients with coexisting pulmonary disease (from 103.0% to 65.0%, *p =*. 031) and in patients without coexisting pulmonary disease (from 91.5% to 87.0%, *p =*. 038).The decrease in the FEV1was more significant in patients with coexisting pulmonary disease than in those without coexisting pulmonary disease (*p =*. 016; S2 Table & S1 Fig). There was no significant difference in the FEV1/FVC change during follow-up between the two groups (*p* =. 79; S3 Table).

#### Respiratory symptoms

The presence of respiratory symptoms was significantly associated with a decreased FVC during follow-up (*p =*. 05; Fig 2B). The median FVC changed from 89.0% to 80.0% in 11 patients with respiratory symptoms (*p =*. 057). In 20 patients without respiratory symptoms, the median FVC changed from 89.0% to 87.0% after RAIT (*p =*. 48).

#### Metastases pattern on chest X-ray

Disseminated pulmonary metastases demonstrated on chest X-ray was associated with a decreased FVC during follow-up (*p* =. 046; Fig 2C). The median FVC of 7 patients who showed disseminated metastases on chest X-ray changed from 91.0% to 70.0% (*p* =. 029). In 24 patients who showed focal metastases or no definite lesion on chest X-ray, the median FVC changed from 88.5% to 87% (*p* =. 79).

#### Progressive disease

The decrease in the FVC during follow-up was also more significant in patients with progressive disease (*p =*. 007; Fig 2D). The median FVC of patients with progressive disease (n = 8) changed from 89.5% to 73.5% (*p =*. 051), whereas the FVC of the other patients (n = 23) changed from 89.0% to 85.0% (*p =*. 42).

#### RAIT

Cumulative I-131 activity was not associated with decreased FVC and FEV1 in serial PFTs (*p* =. 22, *p* =. 47, respectively; S1 and S2 Tables) The median FEV1/FVC of patients treated I-131 > 14.8 GBq changed from 84.0% to 80.0% (*p* <. 001), and the median FVC of patients treated I-131 < 14.8 GBq changed from 80.0% to 75% (*p* =. 77). However, decrease of FEV1/FVC was not associated with cumulative I-131 activity (*p* =. 75; S3 Table). There were also no significant differences in PFT values during follow-up according to the age at first RAIT, sex, smoking history, detection time of metastases, serum Tg level at first RAIT, size of the metastatic lesion (a micro- or macronodular pulmonary lesion), metastases pattern on chest CT, and radioactive iodine uptake pattern on WBS.

### Factors associated with impaired PFT during follow-up by multiple linear regression analyses

Coexisting pulmonary disease was the only significant factor associated with decreased FEV1 and there was no significant factor associated with decreased FEV1/FVC in the single linear regression analysis. Therefore, we performed a multiple linear regression analysis to find the factors associated with decreased FVC during follow-up. Coexisting pulmonary disease and progressive disease were significant factors associated with decreased FVC during follow-up (Table 4).

**Table 4:** Factors associated with decreased FVC by multiple linear regression analysis.

Variables	β ± SE	t	*p*
Coexisting pulmonary disease	15.0 ± 5.6	2.7	.012
Progressive disease	13.0 ± 4.7	2.8	.009

R^2^ =. 39

Respiratory symptom, coexisting pulmonary disease, metastases pattern on chest X-ray, and progressive disease were significant factors associated with decreased FVC in simple linear regression analysis. These factors were included in multiple linear regression analysis, but respiratory symptom and metastases pattern on chest X-ray were excluded in the process of analysis.

## Discussion

The results from this study suggest that mild impairment of baseline pulmonary function, older age, coexisting pulmonary disease, presence of respiratory symptoms, and disease progression of DTC were associated with severe impairment of pulmonary function after RAIT in patients with pulmonary metastases from DTC. To the best of our knowledge, this is the first study evaluating serial pulmonary function to elucidate risk factors for functional impairment after RAIT in adult DTC patients with pulmonary metastases. Neither the number of RAIT nor I-131 activity was associated with changes in pulmonary function after RAIT. This study indicated that pulmonary function in these patients was variable during follow-up after high-dose RAIT. This group contains some high-risk patients whose pulmonary function severely decreases over time.

Distant metastases occurred in 7%-23% of DTC patients [6, 21–24]. The most common metastatic site of DTC is the lung. Even with the metastatic disease, these patients exhibit a longer survival periods than those with distant metastases of other solid cancers. The 10 year overall survival rate of DTC patients with distant metastases vary between 32% and 85% [6–8, 21, 25, 26]. RAIT is thus far the most effective and widely used therapeutic modality for distant metastases of DTC. Physicians sometimes encounter several complications of RAIT during long-term follow-up for patients with metastatic DTC including nausea, vomiting, salivary gland swelling, recurrent sialadenitis, nasolacrimal outflow obstruction, pulmonary fibrosis, and second malignancy in solid organs or bone marrow [27, 28].

Pulmonary fibrosis with impaired pulmonary function after RAIT has been reported in 7% of children or adolescent patients with pulmonary metastases of DTC related to the Chernobyl catastrophe [11]. In contrast, pulmonary fibrosis after RAIT develops in less than 1% of adult patients with pulmonary metastases of DTC [10]. The extent of the damage from radiation is determined by a complex of physical factors, such as the radiation dose, and biological factors, such as the oxygenation of the tissue [29, 30]. Radiation causes more damage in highly oxygenated tissues, and the lungs are thus particularly sensitive to radiation [31]. Radiation pneumonitis is an acute adverse effect of radiation that usually develops within 6 months after exposure to more than 8 Gy of X-rays or gamma rays [30]. Ground-glass opacities within the area of the irradiated lung are seen on radiologic imaging studies in the acute phase. In the chronic phase, dense consolidations or lung volume loss are seen on chest X-ray or CT [32]. In this study, only one patient demonstrated aggravated pulmonary fibrosis after RAIT. However, confirmation of radiation pneumonitis was difficult because she had interstitial lung disease and there was already mild pulmonary fibrosis before RAIT.

In the 1950s, one study reported pulmonary complications of RAIT in thyroid cancer patients with pulmonary metastases [12]. The authors reviewed 15 patients with thyroid cancer with pulmonary metastases, and 6 patients (40%) developed radiation pneumonitis or pulmonary fibrosis. The authors suggested that internal radiation should not exceed 4.625 GBq (125 mCi) in any single dose and that RAITs should be separated by at least a 6-month interval. Benua and Leeper proposed that whole-body retention of I-131 at 48 hours after administration should not exceed 2.96 GBq (80 mCi) in DTC patients with diffuse pulmonary metastases to prevent pulmonary fibrosis and pneumonitis [33]. Our study enrolled DTC patients with pulmonary metastases from 1995 who, as recommended, received the maximally safe I-131 activity, which may explain the lack of pulmonary fibrosis after RAIT and the lack of an association between cumulative I-131 activity and impaired pulmonary function after RAIT.

Samuel et al [14] reported the effect of radioactive iodine on the pulmonary alveolar-capillary membrane using ^99m^TC-DTPA clearance as an index of pulmonary damage in 35 patients with pulmonary metastases of DTC. Six patients with extensive bilateral pulmonary metastases had moderate to severe restrictive pulmonary function. Other studies reported pulmonary fibrosis after RAIT in patients with pulmonary metastases of thyroid cancer [11, 13, 14]. However, these studies did not analyze clinical factors for severely impaired pulmonary function in patients with pulmonary metastases from DTC. Our study demonstrated that mild impairment of baseline pulmonary function, older age, presence of coexisting pulmonary disease, respiratory symptoms, and progressive disease of DTC were significantly associated with decreased pulmonary function on serial PFTs.

This study has some limitations. First, the study is limited by its retrospective design. There were only a small number of study subjects because PFTs have not been routinely assessed in DTC patients with pulmonary metastases. Second, there could have been a selection bias because PFT was usually performed before RAIT when patients had respiratory symptoms or other risk factors for aggravating pulmonary function. Third, it was impossible to calculate I-131 activity absorbed in the lung because we used a fixed-dose approach, as used by other many institutes. Nonetheless, this was the first study to evaluate the risk factors for functional impairment of the lung in patients with pulmonary metastases of DTC before and after RAIT. This study provides clues that PFT should be performed in some high risk patients with pulmonary metastases. Large-sized prospective studies in the future will be able to provide more comprehensive information about the changes in pulmonary function in these patients.

In conclusion, our study revealed that pulmonary function was severely impaired during follow-up in 16% of the patients with pulmonary metastases of DTC after a median of three high-dose RAITs. Neither the number of RAIT nor the cumulative I-131 activity was associated with the changes in pulmonary function after RAIT. However, mild impairment of baseline pulmonary function, older age, coexisting pulmonary disease, respiratory symptoms, and progressive disease of DTC were associated with severe impairment of pulmonary function after RAIT.

## Supporting Information

S1 Fig The changes in FEV1 at baseline and the worst value during follow-up in patients with or without coexisting pulmonary disease. (DOC)

S1 Table The changes in FVC at baseline and during follow-up after RAIT according to clinical factors. (DOC)

S2 Table Changes in the FEV1 at baseline and during follow-up after RAIT according to clinical factors. (DOC)

S3 Table Changes in the FEV1/FVC at baseline and during follow-up after RAIT according to clinical factors. (DOC)
