# Correction: Changes in the Pulmonary Function Test after Radioactive Iodine Treatment in Patients with Pulmonary Metastases of Differentiated Thyroid Cancer

**DOI:** 10.1371/journal.pone.0130220

**Published:** 2015-06-04

**Authors:** The PLOS ONE Staff

The corresponding author designation is incorrect. Won Gu Kim is the correct corresponding author. The publisher apologizes for this error.
