# Supplementary material for: Changes in the Pulmonary Function Test after Radioactive Iodine Treatment in Patients with Pulmonary Metastases of Differentiated Thyroid Cancer
Source: PLoS One. 2015 Apr 29;10(4):e0125114. doi: 10.1371/journal.pone.0125114 (PMC4414613; doi:10.1371/journal.pone.0125114)
Supplement: S1 Fig — (DOC) [file pone.0125114.s001.doc]

**S1 Fig. The changes in FEV1 at baseline and the worst value during follow-up in patients with or without coexisting pulmonary disease**





%, % of measured to predicted values.

FEV1, forced expiratory volume in 1 second; Worst, worst values of pulmonary function during follow-up.
