# Supplementary material for: Changes in the Pulmonary Function Test after Radioactive Iodine Treatment in Patients with Pulmonary Metastases of Differentiated Thyroid Cancer
Source: PLoS One. 2015 Apr 29;10(4):e0125114. doi: 10.1371/journal.pone.0125114 (PMC4414613; doi:10.1371/journal.pone.0125114)
Supplement: S1 Table — (DOC) [file pone.0125114.s002.doc]

**S1 Table 1.** The changes in FVC at baseline and during follow-up after RAIT according to clinical factors.

|  | **FVC (%)** | | ***p**** | ***p***** | **β ± SE** |
| --- | --- | --- | --- | --- | --- |
|  | **Baseline** | **Worst** |  |  |  |
| **Age at first RAIT** |  |  |  | .12 | 7.9 ± 4.9 |
| Age < 45 years (n = 16) | 91.0  (81.5, 101.0) | 91.0  (80.8, 97.3) | .52 |  |  |
| Age ≥ 45 years (n = 15) | 86.5 (75.3, 93.5) | 77.0 (70.3, 83.3) | .051 |  |  |
| **Sex** |  |  |  | .28 | 5.6 ± 5.0 |
| Male (n = 13) | 90.0  (88.0, 95.0) | 85.0 (77.0, 96.0) | .3 |  |  |
| Female (n = 18) | 86.0  (79.3, 93.8) | 85.0 (75.5, 95.8) | .28 |  |  |
| **Respiratory symptoms** |  | |  | **.05** | **10.0 ± 5.0** |
| (-) (n = 20) | 89.0  (80.8, 93.5) | 87.0  (79.8, 96.3) | .48 |  |  |
| (+) (n = 11) | 89.0  (78.5, 95.5) | 80.0 (58.0, 93.0) | .057 |  |  |
| **Coexisting pulmonary disease** |  |  |  | **.009** | **17.2 ± 6.1** |
| (-) (n = 26) | 88.5  (80.3, 94.8) | 87.0 (77.5, 96.0) | .29 |  |  |
| (+) (n = 5) | 91.0  (88.0, 93.0) | 80.0 (46.0, 85.0) | .07 |  |  |
| **Smoking history** |  |  |  | .58 | 3.8 ± 6.9 |
| Never smoker (n = 26) | 87.5  (79.3, 93.8) | 85.0 (75.3, 95.8) | .1 |  |  |
| Smoker (n = 5) | 92.0  (90.0, 97.0) | 84.0 (77.0, 96.0) | .26 |  |  |
| **Baseline pulmonary function** |  |  |  | .37 | 4.9 ± 5.4 |
| Normal (n = 24) | 91.0  (87.0, 95.0) | 91.0  (85.0, 97.0) | .5 |  |  |
| Abnormal (n = 7) | 76.5  (68.8, 89.5) | 74.5 (54.8, 80.0) | .14 |  |  |
| **Serum Tg at first RAIT†** |  |  |  | .4 | 4.9 ± 5.7 |
| ≤ 621 µg/L (n = 23) | 89.0 (82.0, 94.0) | 89.0  (79.5, 96.5) | .4 |  |  |
| > 621 µg/L (n = 8) | 86.5  (75.3, 93.5) | 77.0 (70.3, 83.3) | .15 |  |  |
| **Cumulative I-131 activity** |  | |  | .22 | -7.1 ± 5.6 |
| ≤ 14.8 GBq (400 mCi, n = 12) | 89.0  (79.8, 94.0) | 84.5  (76.8, 97.3) | .31 |  |  |
| > 14.8 GBq (400 mCi, n = 19) | 89.0  (82.5, 94.5) | 85.0  (75.5, 95.5) | .24 |  |  |
| **Size of the metastasis** |  | |  | .25 | 6.1 ± 5.2 |
| Micronodular (n = 20) | 88.5 (80.8, 95.8) | 88.0 (80.0, 96.0) | .39 |  |  |
| Macronodular (n = 11) | 91.0  (83.0, 94.5) | 77.0 (69.5, 93.0) | **.029** |  |  |
| **Metastasis pattern on chest X-ray‡** |  | |  | **.046** | **11.8 ± 5.7** |
| Focal or none (n = 24) | 88.5 (81.8, 94.3) | 87.0  (79.8, 96.3) | .79 |  |  |
| Disseminated (n = 7) | 91.0  (76.5, 94.5) | 70.0 (59.5, 89.5) | **.029** |  |  |
| **Metastasis pattern on chest CT‡** |  | |  | .18 | 6.9 ± 5.0 |
| Focal or none (n = 19) | 88.0 (80.5, 91.0) | 85.0  (79.5, 93.5) | .47 |  |  |
| Disseminated (n = 12) | 93.0 (85.0, 97.8) | 86.5 (69.8, 97.0) | **.019** |  |  |
| **Uptake pattern on WBS** |  | |  | .7 | -2.4 ± 6.1 |
| Focal or none (n = 7) | 88.0 (87.5, 101.5) | 89.0 (73.0, 102.0) | .33 |  |  |
| Diffuse (n = 24) | 89.5 (79.8, 94.3) | 85.0 (77.8, 95.3) | .09 |  |  |
| **Progressive disease** |  |  |  | **.007** | **14.8 ± 5.1** |
| (-) (n = 23) | 89.0  (80.5, 93.5) | 85.0 (80.0, 96.0) | .42 |  |  |
| (+) (n = 8) | 89.5  (84.5, 100.5) | 73.5 (49.0, 95.5) | .051 |  |  |

Unless otherwise indicated, all values are reported as the median (IQR).

^*^ *p* value according to paired *t* test or Wilcoxon signed-rank test comparing PFT values before and after RAIT.

^**^ *p* value according to simple linear regression analyses for comparing changes of pulmonary function during follow-up.

^†^, the 3^rd^ quartile value of serum thyroglobulin at first RAIT was 621 µg/L and we categorized patients according to the serum thyroglobulin level at ablation above or below 621 µg/L.

‡, Five patients showed no disseminated metastatic lesion on chest X-ray, but disseminated metastases with micro-nodules on chest CT.

% in FVC and FEV1 denote the percentage of measured to predicted values. RAIT, radioactive iodine treatment; n, number; Tg, thyroglobulin; CXR, chest X-ray; CT, computed tomography; WBS, whole body scan.
