# Supplementary material for: Changes in the Pulmonary Function Test after Radioactive Iodine Treatment in Patients with Pulmonary Metastases of Differentiated Thyroid Cancer
Source: PLoS One. 2015 Apr 29;10(4):e0125114. doi: 10.1371/journal.pone.0125114 (PMC4414613; doi:10.1371/journal.pone.0125114)
Supplement: S2 Table — (DOC) [file pone.0125114.s003.doc]

**S2 Table. Changes in the FEV1 at baseline and during follow-up after RAIT according to clinical factors.**

|  | FEV1 (%) | | *p** | *p*** | β ± SE |
| --- | --- | --- | --- | --- | --- |
|  | Baseline | Worst |  |  |  |
| Age at first RAIT |  |  |  | .47 | 5.0 ± 6.8 |
| Age < 45 years (n = 16) | 93.5  (87.5, 98.8) | 90.0 (76.8, 95.0) | .15 |  |  |
| Age ≥ 45 years (n = 15) | 87.5  (79.8, 97.5) | 75.0 (67.5, 85.0) | .026 |  |  |
| Sex |  |  |  | .2 | 9.0 ± 6.8 |
| Male (n = 13) | 92.0 (91.0, 98.0) | 87.0 (62.0, 94.0) | .016 |  |  |
| Female (n = 18) | 88.5  (78.3, 103.0) | 86.5 (72.5, 97.3) | .11 |  |  |
| Respiratory symptoms |  |  |  | .93 | 0.6 ± 7.2 |
| (-) (n = 20) | 95.0  (87.5, 98.8) | 90.0 (73.5, 95.0) | .014 |  |  |
| (+) (n = 11) | 84.0  (78.5, 97.5) | 86.0 (57.0, 92.5) | .31 |  |  |
| Coexisting pulmonary disease |  |  |  | .016 | 21.6 ± 8.4 |
| (-) (n = 26) | 91.5  (82.5, 97.5) | 87.0 (74.5, 95.0) | .038 |  |  |
| (+) (n = 5) | 103.0  (78.0, 108.0) | 65.0 (60.0, 77.0) | .031 |  |  |
| Smoking history |  |  |  | .34 | 9.0 ± 9.2 |
| Never smoker (n = 26) | 92.0 (82.3, 101.8) | 87.0 (72.5, 95.0) | .015 |  |  |
| Smoker (n = 5) | 91.0  (82.0, 98.0) | 79.0 (62.0, 93.0) | .13 |  |  |
| Baseline pulmonary function |  |  |  | .85 | -1.4 ± 7.3 |
| Normal (n = 24) | 96.0 (91.0, 103.0) | 93.0 (79.0, 95.0) | .012 |  |  |
| Abnormal (n = 7) | 78.0  (71.3, 81.3) | 65.5  (55.3, 83.3) | .43 |  |  |
| Serum Tg at first RAIT† |  |  |  | .51 | 5.2 ± 7.8 |
| < 621 µg/L (n = 23) | 92.0  (85.0, 99.5) | 87.0  (71.5, 95.0) | .025 |  |  |
| > 621 µg/L (n = 8) | 85.5  (78.8, 97.8) | 75.0  (67.5, 89.8) | .06 |  |  |
| Cumulative I-131 activity |  |  |  | .47 | -5.7 ± 7.8 |
| ≤ 14.8 GBq (400 mCi, n = 12) | 90.0  (78.0, 99.3) | 87.0 (63.5, 96.0) | .06 |  |  |
| > 14.8 GBq (400 mCi, n = 19) | 92.0  (85.0, 99.5) | 87.0 (73.0, 94.5) | .058 |  |  |
| Size of the metastasis |  |  |  | .24 | 8.3 ± 7.0 |
| Micronodular (n = 20) | 93.5  (82.8, 101.5) | 87.0 (76.3, 95.8) | .16 |  |  |
| Macronodular (n = 11) | 89.0  (82.0, 96.5) | 76.0 (60.5, 94.5) | .005 |  |  |
| Metastasis pattern on chest X-ray‡ |  |  |  | .13 | 12.3 ± 7.9 |
| Focal or none (n = 24) | 92.0 (83.5, 98.8) | 87.0 (75.5, 95.0) | .07 |  |  |
| Disseminated (n = 7) | 86.0  (80.0, 100.5) | 66.0 (56.5, 88.5) | .014 |  |  |
| Metastasis pattern on chest CT‡ |  |  |  | .41 | 5.8 ± 7.0 |
| Focal or none (n = 19) | 92.0 (80.5, 97.0) | 87.0 (73.0, 93.5) | .28 |  |  |
| Disseminated (n = 12) | 93.0  (85.0, 103.0) | 86.5 (64.3, 95.8) | .007 |  |  |
| Uptake pattern on WBS |  |  |  | .87 | 1.3 ± 8.2 |
| Focal or none (n = 7) | 103.0 (84.0, 103.5) | 95.0 (64.0, 99.5) | .62 |  |  |
| Diffuse (n = 24) | 91.5  (81.3, 96.5) | 87.0 (73.5, 94.0) | .014 |  |  |
| Progressive disease |  |  |  | .47 | 5.7 ± 7.8 |
| (-) (n = 23) | 92.0  (87.0, 98.0) | 87.0 (76.5, 94.5) | .022 |  |  |
| (+) (n = 8) | 82.0  (76.3, 103.0) | 61.0 (57.8, 98.5) | .37 |  |  |

Unless otherwise indicated, all values are reported as the median (IQR).

^*^*p* value according to the paired *t* test or Wilcoxon signed-rank test comparing PFT values before and after RAIT.

^**^ *p* value according to simple linear regression analyses for comparing changes of pulmonary function during follow-up.

^†^, the 3^rd^ quartile value of serum thyroglobulin at first RAIT was 621 µg/L and we categorized patients according to the serum thyroglobulin level at ablation above or below 621 µg/L.

‡, Five patients showed no disseminated metastatic lesion on chest X-ray, but disseminated metastases with micro-nodules on chest CT.

% in FVC and FEV1 denote the percentage of measured to predicted values. RAIT, radioactive iodine treatment; n, number; Tg, thyroglobulin; CXR, chest X-ray; CT, computed tomography; WBS, whole body scan.
