# Supplementary material for: Changes in the Pulmonary Function Test after Radioactive Iodine Treatment in Patients with Pulmonary Metastases of Differentiated Thyroid Cancer
Source: PLoS One. 2015 Apr 29;10(4):e0125114. doi: 10.1371/journal.pone.0125114 (PMC4414613; doi:10.1371/journal.pone.0125114)
Supplement: S3 Table — (DOC) [file pone.0125114.s004.doc]

**S3 Table. Changes in the FEV1/FVC at baseline and during follow-up after RAIT according to clinical factors.**

|  | FEV1/FVC (%) | | *p** | *p*** | β ± SE |
| --- | --- | --- | --- | --- | --- |
|  | Baseline | Worst |  |  |  |
| Age at first RAIT |  |  |  | .4 | -2.8 ± 3.4 |
| Age < 45 years (n = 16) | 86.5  (82.5, 91.0) | 82.0 (78.8, 83.3) | .002 |  |  |
| Age ≥ 45 years (n = 15) | 87.5 (83.0, 91.0) | 78.5 (75.3, 85.8) | .39 |  |  |
| Sex |  |  |  | .55 | 2.1 ± 3.4 |
| Male (n = 13) | 83.0 (79.0, 87.0) | 80.0 (73.0, 83.0) | .07 |  |  |
| Female (n = 18) | 83.5  (80.3, 89.0) | 79.0 (75.0, 82.0) | .029 |  |  |
| Respiratory symptoms |  |  |  | .1 | -5.8 ± 3.4 |
| (-) (n = 20) | 84.0  (78.8, 89.0) | 79.0 (74.5, 82.3) | < .001 |  |  |
| (+) (n = 11) | 83.0  (80.5, 86.5) | 80.0 (74.0, 85.0) | .77 |  |  |
| Coexisting pulmonary disease |  |  |  | .79 | 1.3 ± 4.6 |
| (-) (n = 26) | 83.5  (79.3, 89.0) | 80.5 (76.5, 83.0) | .022 |  |  |
| (+) (n = 5) | 83.0  (82.0, 84.0) | 73.0 (73.0, 74.0) | .02 |  |  |
| Smoking history |  |  |  | .09 | 7.7 ± 4.4 |
| Never smoker (n = 26) | 83.5  (80.3, 89.0) | 80.0 (75.0, 83.0) | .021 |  |  |
| Smoker (n = 5) | 79.0 (74.0, 84.0) | 73.0 (70.0, 78.0) | .08 |  |  |
| Baseline pulmonary function |  |  |  | .19 | -4.8 ± 3.5 |
| Normal (n = 24) | 84.0  (80.0, 89.0) | 80.0 (75.0, 82.0) | < .001 |  |  |
| Abnormal (n = 7) | 82.5  (71.0, 88.3) | 78.5 (74.0, 83.8) | .9 |  |  |
| Serum Tg at first RAIT† |  |  |  | .29 | 4.1 ± 3.8 |
| < 621 µg/L (n = 23) | 82.0  (78.5, 86.0) | 79.0  (73.5, 82.5) | .017 |  |  |
| > 621 µg/L (n = 8) | 90.0  (83.0, 91.3) | 80.0  (75.8, 89.5) | .13 |  |  |
| Cumulative I-131 activity |  |  |  | .75 | -1.3 ± 3.9 |
| ≤ 14.8 GBq (400 mCi, n = 12) | 80.0  (75.0, 83.8) | 75.0 (72.0, 82.3) | .77 |  |  |
| > 14.8 GBq (400 mCi, n = 19) | 84.0 (81.5, 91.0) | 80.0 (77.0, 83.0) | < .001 |  |  |
| Size of the metastasis |  |  |  | .51 | 1.8 ± 3.5 |
| Micronodular (n = 20) | 83.5  (80.0, 89.0) | 78.5 (73.8, 83.0) | .009 |  |  |
| Macronodular (n = 11) | 83.0  (78.5, 86.5) | 80.0 (76.5, 82.5) | .18 |  |  |
| Metastasis pattern on chest X-ray‡ |  |  |  | .98 | -.1 ± 4.1 |
| Focal or none (n = 24) | 84.0  (80.0, 89.5) | 79.5 (74.0, 82.3) | .002 |  |  |
| Disseminated (n = 7) | 83.0  (78.0, 84.5) | 79.0 (74.0, 85.5) | .54 |  |  |
| Metastasis pattern on chest CT‡ |  |  |  | .93 | -.3 ± 3.5 |
| Focal or none (n = 19) | 84.0 (80.5, 90.0) | 78.0 (73.5, 82.5) | .013 |  |  |
| Disseminated (n = 12) | 83.0  (77.8, 86.3) | 80.5 (75.0, 83.3) | .26 |  |  |
| Uptake pattern on WBS |  |  |  | .6 | 2.1 ± 4.1 |
| Focal or none (n = 7) | 84.0 (80.0, 86.0) | 75.0 (74.5, 78.5) | .3 |  |  |
| Diffuse (n = 24) | 83.0  (79.8, 89.0) | 81.0 (73.8, 83.3) | .013 |  |  |
| Progressive disease |  |  |  | .2 | -5.0 ± 3.8 |
| (-) (n = 23) | 84.0  (80.0, 89.0) | 80.0 (74.5, 82.5) | < .001 |  |  |
| (+) (n = 8) | 83.0  (75.0, 85.3) | 76.5 (73.8, 84.8) | .94 |  |  |

Unless otherwise indicated, all values are reported as the median (IQR).

^*^*p* value according to paired *t* test or Wilcoxon signed-rank test comparing PFT values before and after RAIT.

^**^ *p* value according to simple linear regression analyses for comparing changes of pulmonary function during follow-up.

^†^, the 3^rd^ quartile value of serum thyroglobulin at first RAIT was 621 µg/L and we categorized patients according to the serum thyroglobulin level at ablation above or below 621 µg/L.

‡, Five patients showed no disseminated metastatic lesion on chest X-ray, but disseminated metastases with micro-nodules on chest CT.

% in FVC and FEV1 denote the percentage of measured to predicted values. RAIT, radioactive iodine treatment; n, number; Tg, thyroglobulin; CXR, chest X-ray; CT, computed tomography; WBS, whole body scan.
